# Shrewd Trick of a Dog

**Published:** 1892-05

**Authors:** 


### SHREWD TRICK OF A DOG.

A lively demonstration of canine reasoning occurred at Keeler, last week. A small brown dog with a most intelligent head, familiarly known to the lower country residents as Barney, has been in the habit for a long time past of following the Darwin stage, never missing a trip. Changing the drivers makes no difference; he clings to the route and not the man. On off days he occasionally makes a visit to Cerro Gordo, and doing so recently was set upon and whipped by a dog there. Attached at Boland's store at Keeler is a big, strong dog, that has quite a reputation as a scrapper. On the morning of the next Cerro Gordo trip Barney was noticed playing with the big dog. When the stage started Barney followed, and as his companion seemed averse to going he would run back and play, then forward, and finally persuaded the John L. Sullivan dog into going, too. Arriving at Cerro Gordo, the little dog made a dash at his former vanquisher. John L. "stood in" and the bully was soundly thrashed. Barney wore a broad grin of satisfaction when he returned to Keeler, but he does not visit Cerro Gordo any more.—*Inyo* (*Cal.*) *Independent.*

